# Obstetrics

**Published:** 1898-06

**Authors:** Walter C. Swayne


### OBSTETRICS.

Sir J. Williams[1] has collected 375 cases of **ovarian tumours in pregnancy**. He showed first that ovarian tumour was not more common in women who were, or had been, than in those who were not, and had never been, pregnant; and moreover that, taking statistics of 1000 cases operated on by Sir Spencer Wells and comparing them with the Registrar General's returns, ovarian tumour was more common in the single than the married both as a whole and between various age limits.

As to the question whether repeated pregnancy increased the liability to ovarian tumours, he showed that more than half the reported cases occurred in the two first pregnancies.

He considers, therefore, that (1) ovarian tumour is proportionately more frequent in the single than the married; (2) ovarian tumour is met with less frequently in each successive pregnancy; (3) a large proportion of cases occurred in primiparæ, and in a large number of cases (83 out of 228) the tumour was present before conception, and that hence we are justified in stating that pregnancy has no influence on the production of ovarian tumour.

He next considered whether gestation gives an impulse to the growth of a tumour already present. He showed that as a rule the rate of growth was hastened by pregnancy only in a small number of cases, in some of which the increased rapidity could be attributed to accidental circumstances.

Next, as to the influence of delivery on the rate of growth, he showed that any direct influence caused by delivery in promoting growth of these tumours did not appear to be the rule.

The influence of the tumour on pregnancy, on the other hand, is

[1] *Brit. M. J.*, 1897, ii. 102.

marked, abortion or premature labour occurring in about 19 per cent.; while various accidents, such as twisting of the pedicle, rupture of the cyst, hemorrhage into and suppuration in the cyst, may be caused by gestation.

The part played by the growth in labour is mainly mechanical: a large growth impedes the auxiliary forces and interferes with cardiac and respiratory action if situated in the abdomen, while if situated in the pelvis it presents a formidable obstacle to delivery, and is the chief cause of the high mortality (25 per cent.) following this complication. The diminution of the enormous mortality can only be effected, in his opinion, in two ways: (1) by the removal of the tumour during pregnancy or labour; (2) by Cæsarean section. Small tumours not occupying the pelvis, and those capable of being pushed up into the abdominal cavity, might be left, and would probably cause few symptoms; all others should be operated on, and Cæsarean section reserved for cases in which the tumour was found to be impacted in the pelvis by strong adhesions.

Austin Cheney[1] reports a case of double ovariotomy during pregnancy, and remarks, in opposition to Williams, that the effect of pregnancy on the tumour is to cause rapid growth, especially when the cyst is small and pelvic in origin. He mentions that Remy states that in 321 cases collected by him, abortion occurred 55 times. This is a higher proportion than that given by Williams. At the same time, he states that the existence of an ovarian tumour with pregnancy is of comparative rarity, and remarks on the dangers to the patient caused by accidents to the tumour, and also from intestinal obstruction caused by pressure.

He points out the risks arising from traction on the pedicle and stretching of adhesions. He quotes Gordon's[2] collection of 204 ovariotomies at different periods of gestation: in 21, where the mothers all recovered, the influence on labour is not stated; in 7 cases the uterus was injured, causing death twice.

Of the remaining 176 cases, 93.2 per cent. recovered, and 6.8 died: in 69.4 per cent. labour took place at term, while in 22 per cent. premature labour followed the operation; but as premature termination of pregnancy occurred in 17 per cent. of the cases quoted by Remy, and in 12 per cent. of those quoted by Williams, when no operation was performed, the tendency to miscarriage after operation does not seem so serious; while if to this is added the enormous fœtal mortality of cases not operated on, the chances of a normal labour at term and of a living child are greatly increased by operation and removal of the growth during pregnancy. As compared with the results either of unassisted labour, or labour where various obstetric operations have to be performed to effect delivery, the mortality after ovariotomy during pregnancy is not so much higher than

[1] *Am. J. Obst.*, 1897, xxxv. 265.
[2] *Centralbl. f. Gynäk.*, 1894, xviii. 566, quoted by Cheney.

that in the non-pregnant condition as to make us consider the operation one of very great risk: Gordon reports a mortality of 6.8 out of 204 cases operated on.

The author narrates an interesting case in which a patient treated for abortion had an ovarian cyst of each ovary; after curetting, she became pregnant, and was operated on during the fifth month of pregnancy and both the tumours removed, a good recovery following. On examination, the larger tumour, which a fortnight before the operation was as large as an adult head and had diminished in size by the date of the operation, was found to have a minute orifice in its wall, through which fluid had escaped into the abdominal cavity. The cyst was papillomatous. The patient went to term and was delivered of a living male child, weighing twelve pounds. Delivery was assisted by forceps to prevent injury to the abdominal scar.

The **obstacle to labour caused by** the presence in the pelvis of **an ovarian tumour** is one of the most serious complications with which the accoucheur can be confronted,—the mother's life being endangered, firstly, by the prolongation of delivery, the risk of rupture of the uterus, and the certainty of serious pressure on the tissues in their normal situation; and secondly, by the risks produced (even when delivery is possible) by the pressure of the fœtus on the tumour, with the possibility of its bursting and discharging its contents into the peritoneal cavity; of its subsequent sloughing from prolonged pressure, or of suppuration of its contents after injury. The death of the fœtus from prolonged uterine action is also very probable, as is the necessity for its destruction by sacrificial operations. Tumours occupying this position are very likely by their pressure on the rectum to cause damage by bruising the walls of the bowel, and subsequent bacterial infection by the bacterium coli, with the possibilities of peritonitis or suppuration in the tumour, if cystic.

The same subject is very fully dealt with by McKerron,[1] who has collated a large number of cases of this complication. He directed his attention solely to those cases where the presence of the tumour was detected after the onset of labour, and the tumour was found to occupy the pelvic cavity. The results of his investigations are very striking, the mortality following this complication being no less than 31 per cent. in all recorded cases. The mortality has, however, diminished very largely during the last twenty years: 48 cases of this complication have been recorded, with a maternal mortality of 6, or 12.5 per cent., and a fœtal mortality of 36 per cent. The diminution of the mortality in the case of both mothers and children is nearly the same, and although one would at first sight attribute this to improved antiseptic measures, this is only likely to affect the maternal

[1] *Tr. Obst. Soc. Lond.*, 1898, xxxix. 334.

mortality, and the diminution of the fœtal mortality should evidently be more properly referred to the earlier commencement of appropriate treatment. He shows that in 34 of the later cases the duration of labour averaged 24 hours; while of 70 of the 135 earlier cases the average duration was 40 hours, showing the advantage of the abandoning of the expectant plan of treatment. Rupture of cyst occurred in 15 cases, death resulting in 9; of 5 cases in which spontaneous rupture of the cyst occurred, 2 were fatal; in 3 cases rupture occurred during reposition, with 2 deaths; in 1 case during vaginal examination, with a fatal result; in 4 cases during extraction by forceps or version, all of these were fatal; and in 2 during craniotomy, with 1 death.

A certain amount of interest attaches to the rate of mortality in patients suffering from each particular variety of tumour, thus:—

| | Cases. | Deaths. | Rate per cent. |
|---|---|---|---|
| Tumours described as cystic ... | 49 | 10 | 20 |
| ,, ,, colloid cysts | 3 | 1 | 33 |
| ,, ,, dermoid | 46 | 18 | 39 |
| ,, ,, malignant | 3 | 3 | 100 |
| ,, ,, carcinoma | 5 | 4 | 80 |
| ,, ,, fibroma | 5 | 4 | 80 |
| ,, ,, fibro-cyst | 1 | 1 | 100 |
| ,, ,, cysto-sarcoma | 1 | 1 | 100 |

Rupture of the cyst occurred 8 times, with 3 deaths. The duration of labour averaged between 40 and 50 hours in 7 of the fatal cases in which it is recorded and 1 is recorded as "very protracted." In 11 cases which recovered, the duration of labour averaged between 20 and 25 hours. Hence, of cases where labour was terminated by the natural powers, the mortality would appear to be greater in dermoids than in cystic tumours, to be in direct ratio to the duration in time, and to be seriously complicated by rupture of the cyst.

Thirty-five cases occurred in which labour was left to the natural powers; of these, 19 are described as cystic, 1 as a colloid cyst, 6 as dermoids, 2 as carcinoma, 2 as malignant. Of the 19 cases in which the tumour is described as cystic, 4 died, or 21 per cent.; of the 6 described as dermoid, 3 died, or 50 per cent.; both those described as malignant ended fatally, as did one of the two described as carcinoma. Forty-one cases are described where reposition was effected before delivery, and of these 6 died; but 3 of these deaths were due to other causes. Forty-three cases were treated by puncture or incision; 7 of these died. The tumour is described as dermoid in 3; in 1 of the cases death was due to eclampsia, so that being excluded, the mortality appears to be 6, or 14 per cent. Seventeen cases were treated by version—of these 6 died, or 30 per cent.; and 14 were delivered by forceps, and of these 8 died, or 57 per cent.; in 4 of these rupture of the cyst occurred, and in 1 twisting of the pedicle. Eighteen cases were treated by craniotomy; of these 8 died, or 44 per cent. Three of the cysts were dermoid, 1 a fibroma, and 1 malignant. Ten cases were delivered by Cæsarean section; of these 8, or 80 per cent., died; but it should be noted that the 2 recoveries occurred in cases operated on since 1894. Three of the cysts were dermoid, 2 were fibromata, 1 a cysto-sarcoma, 1 a colloid cyst, and 1 carcinoma. Two cases were treated by abdominal ovariotomy, both recovered; and 3 by vaginal ovariotomy, and all recovered; in 1 of the cases of abdominal ovariotomy the cyst was found to be colloid, and in 1 of the cases of vaginal ovariotomy, a cystic adenoma was found.

Hirst,[1] reports a case of obstruction of labour by a dermoid cyst in the pelvis. He saw the patient after she had been in labour more than 4 days, and found on examination the whole pelvis blocked by a firm tumour, so that there was not room to pass a finger between the symphysis and the tumour; he found it impossible to displace the tumour, and performed Cæsarean section; he then found it necessary to remove completely the uterus, which was in a gangrenous condition, and the tumour, which proved to be a dermoid cyst of the ovary. Mother and child both did well.

McKerron shows that although the mortality of the later cases has markedly diminished, it is still appallingly high. Reposition of the tumour should always be attempted where possible, but forcible efforts should be avoided. Should reposition fail, the most successful method of treatment, except in the hands of skilled operators, will be found to be puncture or incision; the danger of general peritoneal infection is always present, however, but with modern methods is much less, especially if the cyst walls after incision are sutured to the vaginal wall. Forceps and version should be abandoned altogether, since the mortality with these methods is only less than that of Cæsarean section; craniotomy should not be depended on, as it is subject to the same objections. Cæsarean section appears from the table to be a most fatal procedure, but the mortality of this operation, even when uncomplicated, was extremely high up to 1876, and may be expected in the future to give a much larger number of successes; the last 3 cases recorded, 1 in 1889 and 2 in 1897, all recovered.

The removal of the obstacle by abdominal section has been tried but seldom; the results, however, seem to promise well in the few cases recorded, and are likely to be superior, if the operation is undertaken before exhaustion has occurred, either to those of leaving the case to nature, or to those of puncture or incision.

Vaginal ovariotomy also has been tried in only 3 cases,

[1] *Am. J. Obst.*, 1897, xxxv. 790.

but the successful result of these is encouraging; there seems no good reason why further successes should not attend the use of this method. The importance of early treatment and the necessity for distrusting the natural powers cannot be too strongly insisted on.

Gottschalk[1] states that it is becoming comparatively rare to meet with **ovarian tumours in the puerperal state,** owing to the greater readiness with which ovariotomy is performed nowadays, on the discovery of the tumour during pregnancy, and that only those cases in which the tumour has escaped notice during pregnancy come under the care of the surgeon during the puerperal state—that is, excepting those in which the tumour has been purposely left. The fatality attending the old methods of treatment can be traced, either directly or indirectly, to septic changes in the cyst and its contents, or to rupture of the cyst.

Remy[2] lost 24 women out of 52 cases of rupture. Suppuration and decomposition in the cyst are most frequent after puncture, owing to the introduction of septic matter with the trocar. Undisturbed pregnancy, parturition, and childbed probably only occur in those cases where the tumour is small or of medium size, its walls thick, and its rate of increase only slow. Now, the question is, granted that pregnancy, parturition, and childbed have been uneventful and to all appearances normal, what is the condition of the tumour, and how is it affected by these processes apart from the obvious clinical manifestations, such as rapid growth, and other gross pathological changes?

The gynæcologist learns on the operating table that these apparently normal and undisturbed processes, parturition, etc., before mentioned do not pass without serious alterations in the condition of the tumour.

Löhleim[3] found in 1 case out of 7 he reports that adhesions to intestine were so strong that he was compelled to content himself with partial resection of the cyst, and the enclosure of the remainder in the abdomen. Twisting of the pedicle is very frequent in childbed, due partly to the sudden lowering of intra-abdominal pressure by the rapid evacuation of the contents of the uterus, and partly to the return to its normal position of the deflected uterus, which, apart from the presence of any tumour, tends to be slightly twisted on its long axis.

Suppuration in the cyst, apart from puncture, is another fertile cause of trouble, and by leading to peritonitis may easily cause a fatal result.

He considers that in general, pregnancy and parturition have a bad effect on ovarian tumours in the ways named, and he thinks that trouble nearly always arises during the puerperal

[1] *Samml. klin. Vortr.*, 1898, No. 207.
[2] *Thesis Agr.*, Paris, 1886, quoted by Gottschalk.
[3] *Ztschr. f. prakt. Aerzte*, 1896, v. 199; quoted by Gottschalk.

period if these complicate pregnancy and labour. Other observers do not attach such importance to this complication. Dakin[1] and Williams[2] both state that if small and not in the pelvis, they may be left with safety; bad results should always be anticipated if labour occurs in a case where the tumour is large, low enough in the abdomen to deflect the presenting part, or small and in the pelvic cavity.

WALTER C. SWAYNE.

### Reviews of Books.

[1] *Handbook of Midwifery*, 1897. [2] *Loc. cit.*

